# Unusual trichobezoar of the stomach and the intestine: a case report

**DOI:** 10.1186/1752-1947-8-79

**Published:** 2014-02-28

**Authors:** Issam S Al-Janabi, Muthanna A Al-Sharbaty, Marwan M Al-Sharbati, Laith A Al-Sharifi, Allal Ouhtit

**Affiliations:** 1 Department of Surgery, Baghdad Teaching Hospital, Baghdad, Iraq; 2 Al-Jumhori Teaching Hospital, General Surgery, 2nd Surgical Unit, Mosul, Iraq; 3 Department of Behavioral Medicine, Sultan Qaboos University, Muscat, Oman; 4 Karbala Health Directorate, Karbala, Iraq; 5 Department of Genetics, College of Medicine, Sultan Qaboos University, Muscat, Oman

**Keywords:** Bezoar, Endoscopy, Iraq, Laparoscopy, Mass, Rapunzel syndrome, Trichobezoar, Trichotillomania

## Abstract

**Introduction:**

Trichobezoars are an infrequent form of bezoar found in the stomach or intestine, created from ingested hair. This condition has been well described in the surgical literature, but less reported in psychiatry.

**Case presentation:**

We describe the case of an 18-year-old Middle Eastern Caucasian woman with trichotillomania who presented to our emergency department with a history of central abdominal pain associated with vomiting and constipation for five days. An examination showed a trichobezoar requiring emergent surgical intervention, and indicating the need for psychiatric treatment. The trichobezoar was treated successfully by laparotomy.

**Conclusion:**

The medical and psychiatric sequelae of trichotillomania should not be underestimated, and early diagnosis and treatment is of utmost importance to save the patient’s life and prevent recurrence. Although laparotomy is still considered an excellent option, pharmacotherapy and behavioral assessment play a useful role in patient management. Our case highlights the fundamental concept of a holistic approach rather than only treating the symptoms, by considering factors such as genetic influences to understand the disease.

## Introduction

A trichobezoar is a rare medico-surgical condition consisting of a hair ball in the proximal gastrointestinal tract, which may cause obstruction, that almost exclusively affects young women [1,2]. It results from trichotillomania, a psychiatric disorder characterized by the compulsory and persistent pulling out of one’s hair, involving the hair of the scalp, eyebrows, eyelashes or elsewhere in the body, which leads to noticeable hair loss. The majority of people with this disorder have emotional problems (depression, anxiety) and poor self image; the patient usually suffers from tension prior to pulling, or when trying to resist the action, and subsequently feels pleasure and gratification in doing so [3]. The prevalence rate varies from 0.06% to 4% [4].

When ingested, because of its smooth surface, human hair resists both digestion and peristalsis, and accumulates between the mucosal folds of the stomach. Continuous ingestion of hair over a period of time leads to the impaction of hair together with mucus and food, causing the formation of a trichobezoar. In most cases, the trichobezoar is confined within the stomach. In some cases, however, the trichobezoar extends through the pylorus into the jejunum, ileum or even colon. This condition is called Rapunzel syndrome and was first described by Vaughan *et al.* in 1968 [5]. In addition, parts of the tail can break off and migrate to the small intestine, causing intestinal obstruction [6-8]. Trichobezoars may not be recognized in their early stages because of their nonspecific presentation, or even lack of symptoms. Following the introduction of minimally invasive surgery and endoscopy with mechanical and laser fragmentation techniques, some authors have questioned the necessity of a laparotomy to treat trichobezoars, and consider these new techniques more convenient for trichobezoar removal [7-9].

Establishing a pedigree with family and social history can ultimately help in the differential diagnosis. We describe a case of trichotillomania in a young woman that led to the formation of a trichobezoar that needed emergent surgical intervention and follow-up psychiatric treatment. We highlight the fundamental concept of treating the whole person rather than just symptoms by considering factors such as genetic influences in understanding the disease.

## Case presentation

An 18-year-old single Middle Eastern Caucasian woman presented to our emergency department with a history of central abdominal pain, colicky in nature, associated with vomiting and constipation for five days. No other complaints were reported. She was, however, admitted to hospital 18 months previously for the same complaint, and treated conservatively as a case of sub-acute intestinal obstruction. She lived with her parents, had no evident psychiatric illness, drug history or known allergy, and had no prior surgical history.

A clinical examination revealed that our patient was fully conscious, mildly dehydrated and neither pale nor jaundiced. Her body weight, heart rate, blood pressure and respiratory rate were all within normal ranges. She had an obvious asymmetrical abdominal distension with a centrally inverted umbilicus, and no scar was observed. Her abdomen was soft on palpation, with a left hypochondrial intra-abdominal immobile mass, extending to her epigastric region, measuring about 16cm × 10cm. Another mobile oval mass was also seen in her left iliac fossa, measuring about 8cm × 5cm. Both masses had well-defined round edges and smooth surfaces, and were not tender, compressible or pulsatile. Her bowel sounds were exaggerated and high pitched, and both her rectum (by digital examination) and hernia orifices were empty. Laboratory results showed the following: packed cell volume, 36%; white blood cells, 8×10^9^ cells/L; erythrocyte sedimentation rate, 16mm/hour; blood urea, 6.2mmol/L; serum creatinine, 110μmol/L; serum potassium, 4.2mmol/L; serum sodium, 138mmol/L.

**Figure 1:**
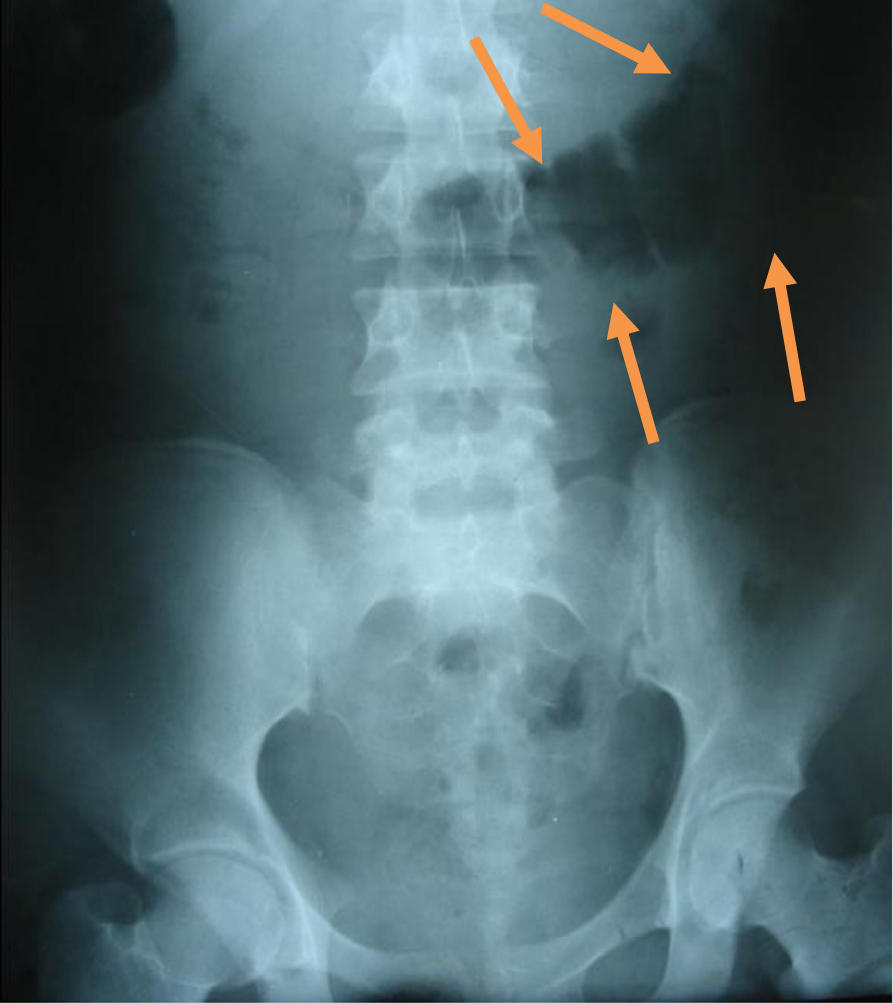
Plain X-ray of the abdomen showing a well-defined, rounded soft tissue density mass, in the central abdominal region with calcification (arrows).

**Figure 2:**
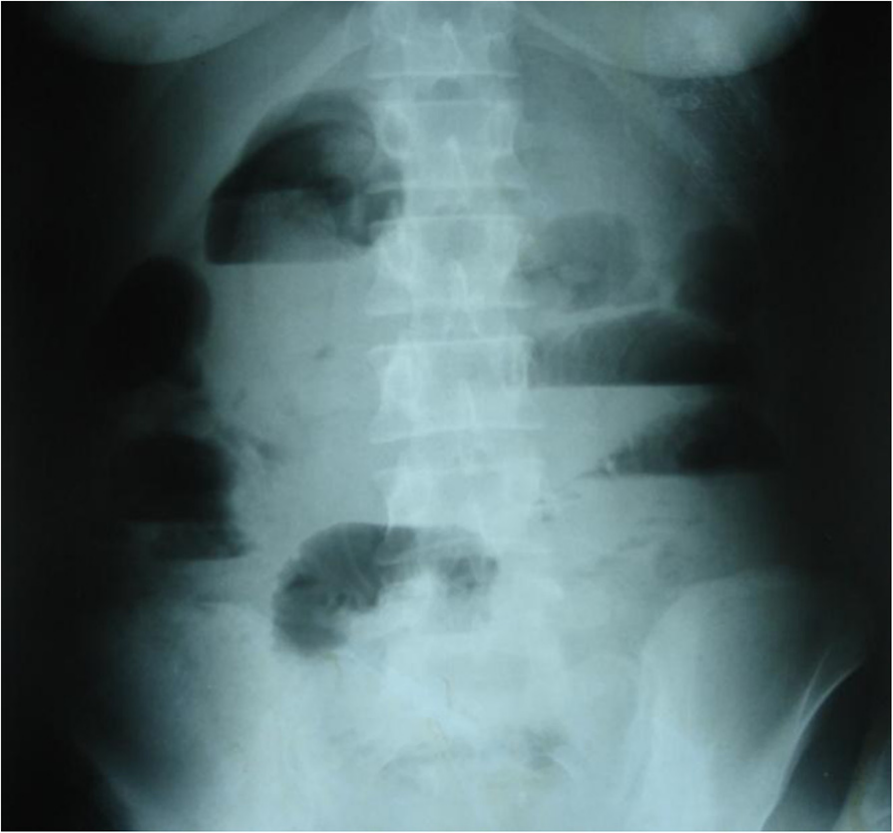
**Upright plain abdominal X-ray demonstrating a small bowel obstruction.** Note the presence of multiple air fluid levels.

An abdominal X-ray showed a well-defined, rounded soft tissue density mass in her central abdominal region, with calcification (Figures 1 and 2). An abdominal ultrasound showed a central abdominal oval lesion, with peripheral calcification and gaseous bowel distension; the lesion was not related to her liver or spleen. A computed tomography scan was not performed as it was not available in our emergency department.

**Figure 3:**
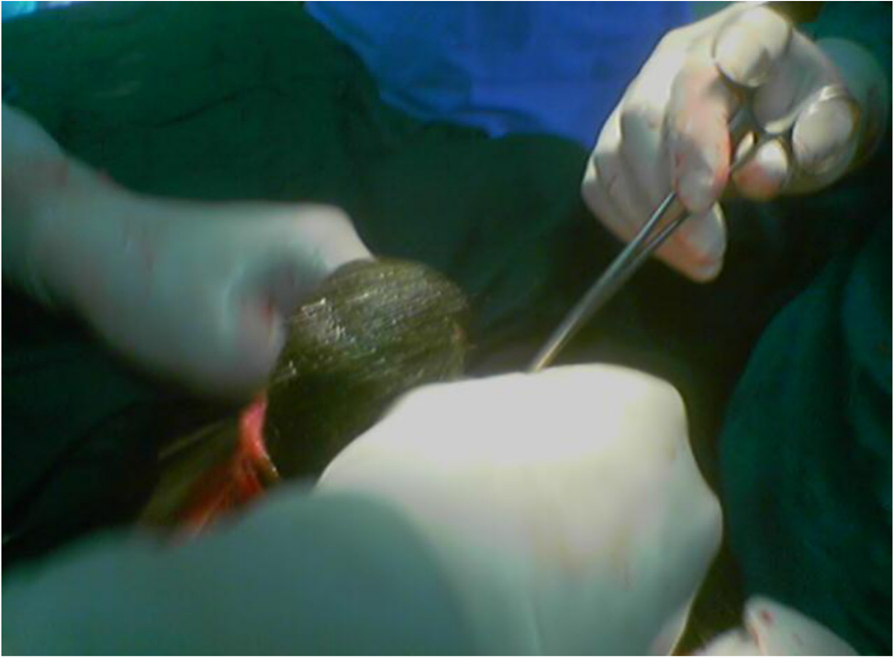
Removal of the trichobezoar from the stomach.

**Figure 4:**
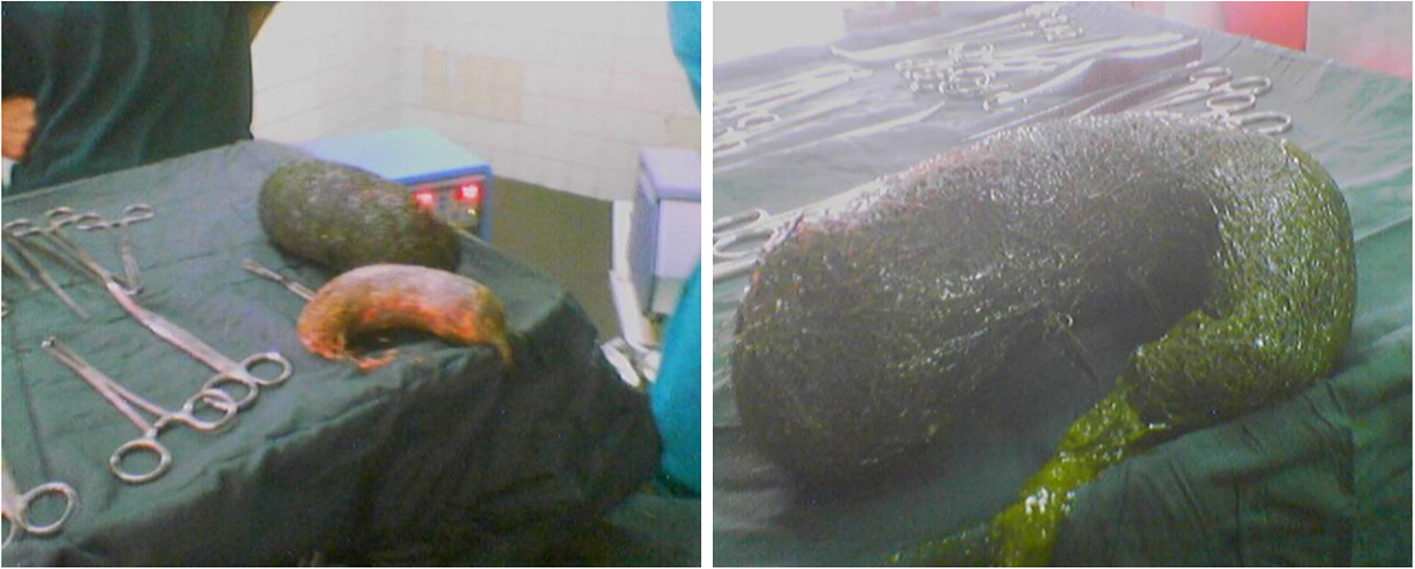
Removal of trichobezoar revealed two masses taking the shape of the stomach and ileum.

Conservative treatment was initiated by stopping oral feeding, placing a nasogastric tube, and starting intravenous fluids. Our patient received antibiotic treatment (cefotaxime 1g and metronidazole 500mg) and was put under observation. Her condition deteriorated, with her temperature reaching 37.8°C 12 hours after admission, and her pulse rate increasing to 120 beats/minute, although her blood pressure remained within normal range (110/70mmHg). A physical examination of her abdomen showed mild tenderness in her lower abdomen, and ‘tinkling’ bowel sounds. The nasogastric tube collected 300cm^3^ of watery fluid over 12 hours. Explorative laparotomy identified two masses, one in her stomach and another in her ileum. We used a combination of gastrotomy and enterotomy to remove the trichobezoars from both her stomach and the ileum (Figures 3 and 4). The closure was performed by double layers (inner absorbable 2/0 vicryl and outer 2/0 silk).

## Discussion

Although rare, a trichobezoar may present as an emergency that requires proper preparation by the surgeons. Due to the unavailability of endoscopy and computed tomography scan facilities in our emergency department to diagnose the case pre-operatively, we depended on a high index of suspension in the management of our patient. We performed an explorative laparotomy as an emergency operation to save our patient’s life.

We collected all case reports or studies of trichobezoar in children and adolescents published during the last 20 years using the MEDLINE search tool, with ‘trichobezoar’ as the keyword. During the last few years, trichobezoar cases have attracted debate about the application of minimally invasive techniques, such as endoscopy and laparoscopy [7-14], rather than laparotomy, as well as medical treatment and enzymatic degradation, which are attractive because of their noninvasiveness but have been reported to be ineffective [15,16].

Endoscopic removal, if successful, would be considered the most attractive treatment option. The first report of the successful endoscopic removal of a trichobezoar was for one that was relatively small, weighing only 55g [17]. Reports of successful endoscopic removal of trichobezoars in children are remarkably scarce - they are vastly outnumbered by case reports documenting unsuccessful attempts of endoscopic removal with or without fragmentation [8,14]. In our patient, two big masses were found, and endoscopic removal would have been challenging and not safe.

An analysis of the published case reports revealed that out of 40 cases in which endoscopic removal had been tried, only two (5%) were successful [8]. In one of these, a trichobezoar was successfully removed whole from the distal esophagus [8]. In a series of 15 patients with bezoars, a 15-year-old girl underwent fragmentation of a large trichobezoar by means of a modified needle-knife and mono-polar coagulation current. In most case reports, however, fragmentation was considered impossible because of the size, density and hardness of the mass, and endoscopy was not considered a viable therapeutic option [7,14]. Moreover, because the removal of all fragments requires repeated introduction of the endoscope, pressure ulceration, esophagitis and even esophageal perforation may occur [9]. Also, fragments of a large trichobezoar might migrate through the pylorus after fragmentation or repeated manipulation, causing intestinal obstruction. Careful examination of the intestine for satellites cannot be performed by endoscopy, and the removal of those fragments is impossible. Although not a therapeutic option, endoscopy may prove to be extremely valuable as a diagnostic modality in patients in whom the nature of the gastric mass is unclear. It enables the differentiation between trichobezoars and foreign bodies that can be fragmented and removed using endoscopy [18].

In one study, a laparoscopy was used for the initial procedure but then converted into a laparotomy when difficulties were encountered as a result of a large intragastric mass. In some centers, laparoscopy is considered inferior to laparotomy for the treatment of a trichobezoar. Nirasawa *et al.*[10] were the first to report on laparoscopic removal of a trichobezoar. Since then, only six other reports of attempted laparoscopic removal have been published [7-9,11-13]. The lack of reports on endoscopic treatment might partly be explained by the rarity of trichobezoars, but it could also indicate that laparoscopy is not an attractive treatment modality for trichobezoar. Of the six case reports, two reported failure to remove the trichobezoar, which was attributed to the large size of the trichobezoar and to the presence of satellite trichobezoars in other locations of the gastrointestinal tract (as in our patient) [13,14]. In one study, endoscopic and laparoscopic approaches were combined; because endoscopic fragmentation of the bezoar was not possible, a laparoscopic approach was used to fragment the trichobezoar, then endoscopy used to remove the fragments [19]. Successful laparoscopic removal, however, requires a significantly longer operation time compared to conventional laparotomy, mostly due to the complexity of the operation. Careful examination of the entire digestive system (stomach and intestine) is necessary to avoid secondary intestinal obstruction due to satellites. With laparoscopy, this procedure is far more challenging; the risk of spilling contaminated hair fragments into the abdominal cavity makes the laparoscopic approach even less desirable. In addition, the rarity of trichobezoars makes it difficult to achieve a good technique for laparoscopic removal and inspection of the entire intestine. However, the laparoscopic removal of trichobezoars with intestinal obstruction has advantages compared to laparotomy, including better cosmetic outcome, fewer postoperative complications and reduced admittance time [20]. Though several reports stress the excellent cosmetic result of the laparoscopic approach, they also report the frequent need to extend the initial port wounds, sometimes by up to 4cm [7,10].

A laparotomy was successful in most cases of trichobezoars, including our case. In the literature, the cases of 100 patients who underwent successful conventional laparotomy were identified. However, 12% had one or more complications, including perforation of the intestine during removal of the trichobezoar [21,22], minor wound infection [23], pneumonia, paralytic ileus [24], and ileal trichobezoar and fecal leakage through the lower part of the laparotomy wound [25]. Due to the high success rate, and the relatively low complication rate, the low complexity, and the ability to carefully examine the entire gastrointestinal tract for satellites in a short period of time, laparotomy is still considered the treatment of choice in our center.

The literature provides no evidence of superiority of endoscopy or laparoscopy over laparotomy. The lack of invasiveness of these techniques does not seem to outweigh the disadvantages and the complexity of these procedures.

In addition to the acute surgical treatment for a trichobezoar, psychiatric consultation is crucial to prevent relapses and to treat comorbid conditions that usually accompany this disorder. Trichotillomania followed by swallowing the hair (trichophagia) is considered to be one type of pica, which is defined as ‘the persistent craving and compulsive eating of non-food substances’, such as hair, sponge, soap, sand and so on [26]. However, other comorbid psychiatric disorders, in which genetic factors might play a role, are also associated with trichobezoar (for example, obsessive compulsive disorder, depression and anorexia nervosa) [27,28]. For these reasons, psychiatric referral after surgical treatment of a trichobezoar must be considered as an essential part of successful treatment and prevention of recurrence. Therefore, behavioral treatment appears to be the priority measure, even with patients older than age 16 years [28]. Although not all psychiatrists agree to adopt pharmacotherapy, it may be used temporarily to treat accompanying disorders (for example, serotonin reuptake inhibitors). Unfortunately, the benefit of such treatment is not consistent, but the combination of such treatment with psychotherapy appears to be effective. After improvement and discharge from the hospital, our patient was referred to a psychiatrist for appropriate psychological intervention and follow-up.

## Conclusions

The medical and psychiatric sequelae of trichotillomania should not be underestimated, and early diagnosis and treatment are of utmost importance to save the patient’s life and prevent recurrence. Although laparotomy is still considered an excellent treatment option, pharmacotherapy and behavioral assessment play a useful role in patient management.

Our experience with this patient highlights the fundamental concept of treating the whole person rather than just symptoms by considering factors such as genetic influences to understand the disease.

## Consent

Although our patient was of legal age, according to the customs of Arab countries, written informed consent was obtained from the patient’s parent for publication of this case report and any accompanying images. A copy of the written consent is available for review by the Editor-in-Chief of this journal.

## Competing interests

The authors declare that they have no competing interests.

## Authors’ contributions

ISA conceived of the study, participated in its design and coordination, and helped to draft the manuscript. MAA participated in the design and coordination of the case, and carried out the literature search. MMA participated in the interpretation of the case and the writing of the manuscript. LAA participated in the coordination and interpretation of the case. AO contributed to drafting the manuscript. All authors read and approved the final manuscript.
